# The peer review gap: A longitudinal case study of gendered publishing and occupational patterns in a female-rich discipline, Western North America (1974–2016)

**DOI:** 10.1371/journal.pone.0188403

**Published:** 2017-11-29

**Authors:** Shannon Tushingham, Tiffany Fulkerson, Katheryn Hill

**Affiliations:** 1 Department of Anthropology, Washington State University, Pullman, Washington, United States of America; 2 Department of Anthropology, University of California Davis, Davis, California, United States of America; Iowa State University, UNITED STATES

## Abstract

Researchers have repeatedly demonstrated that women continue to be underrepresented in publication output in the sciences. This is true even in female-rich fields such as archaeology. Since most gender-related publication studies rely on data from peer-reviewed journals, it would be instructive, though challenging, to also track publication output in non-refereed and professional or industry venues, which tend to be more accessible to those working in extra-academic settings. This comparison is important in fields such as archaeology in which the vast majority (approximately 90%) of practitioners in the USA work for private sector cultural resource management firms and federal and state agencies. To understand the dynamics of who publishes where, we compiled a new dataset tracking over 40 years of peer-reviewed versus non-peer-reviewed publications that publish articles on the archaeology of California (an American Indian cultural area including southwest Oregon, most of the state of California, and Baja Mexico) and the Great Basin culture area (spanning eight western USA states). Historic gender differences in the publishing output of authors identified as men versus those identified as women were revealed by articles published between 1974 and 2016 in two refereed journals, the *Journal of California Anthropology*/ *Journal of California and Great Basin Anthropology* and *California Archaeology*, and in one un-refereed venue, the Society for California Archaeology *Proceedings*. Although multiple independent measures indicate that women are contributing and active members of the discipline, publishing records yield more variable results. Specifically, while women have historic and increasingly robust levels of participation in the non-peer-reviewed *Proceedings*, they remain vastly underrepresented in the two peer-reviewed journals, which are widely regarded as more prestigious and influential. We argue that this “peer review gap” is influenced by variation in the costs (largely time investment) and benefits of publication for people working in different professional roles (e.g., agency professionals, private/cultural resource management firm personnel, tenure-track faculty, adjunct faculty, etc.). We also argue that these cost and benefit variations may ultimately influence the decisions of people of all genders and backgrounds, but, because of the current structure of our discipline—including the fact that women and minorities lag in positions where costly peer-reviewed publication is a rewarded and supported activity—overwhelmingly affect these groups. We recognize that non-refereed publications such as *Proceedings* provide an important means of bridging the peer review gap and give voice to individuals from diverse backgrounds and perspectives.

## Introduction

**Fig 1:**
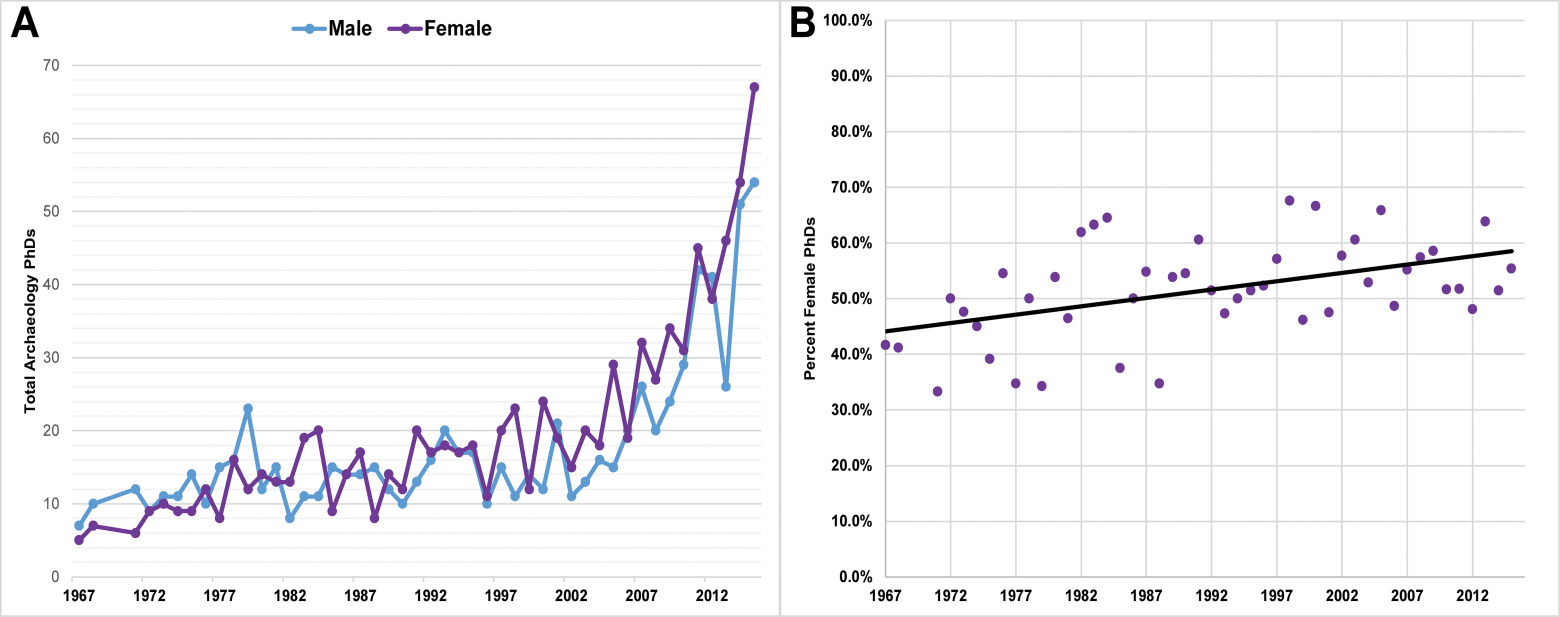
(A) Total number of archaeology PhDs earned in the USA, and (B) Percentage of PhDs earned by women in the USA. SOURCE: National Science Foundation, National Center for Science and Engineering Statistics, 2015 Survey of Earned Doctorates; special tabulation (May 2017). Note: 1969 and 1970 data not available.

There remain persistent gender inequities in the publication output of men versus women researchers in the sciences [1–3]. This is true even in disciplines that are otherwise “female-rich,” or those that currently have equal or greater numbers of women participants [1, 4]. Archaeology is one such field. While in the early years of the discipline there were far more male practicing archaeologists in the USA, today women account for more than half of PhDs awarded in archaeology (Fig 1), and, as of 2013, women comprise 47% of the 7,391 members of the Society for American Archaeology (SAA), a figure that reflects the roughly equal proportion of men and women in the field of American archaeology [5]. Despite gender parity in overall composition, a number of studies have demonstrated that there is a persistent gender gap in certain aspects of the discipline, including reduced pay scale for women [6], lower hiring rates of women in academic settings in proportion to their representation among PhD recipients [7–9], lower numbers of women in top-tier tenure-track academic positions [6, 10], lower success rates of grant proposals for junior female researchers [11], and overrepresentation of women filling non-prestigious/ managerial roles in the public and private sector [12]. Publication rates, widely taken as a direct measure of scientific productivity, have also largely remained low for women [5, 10, 13, 14]. While there are encouraging trends—for example, women’s publication rates in *American Antiquity*, the SAA’s flagship peer-reviewed journal, rose from 5% of lead authors between 1967–1991 [14], to 24.0% between 1990–2013 [5]—the publication gap persists: a recent survey of five national and international research journals dating between 1990–2013 found that an average of 28.6% of papers were written by women, and a similar proportion of papers (28.7%) in six regional journals from the USA had female lead authors [5].

Reasons for the gender gap are complex and may include authorial behavior [5] along with work/ familial obligations that influence such behavior [15–17], editorial or reviewer bias [18–22], and mentorship [23–25], as well as structural aspects of archaeology and other disciplines. In publication studies of gender, publication rate—a common benchmark used to assess scientific productivity—is almost exclusively measured by the number of peer-reviewed papers an author publishes. However, some researchers have questioned whether publication rate is a relevant or unbiased metric to begin with [26] and whether peer-reviewed publications should be treated as the “gold standard” of professional achievement at the expense of extra-academic venues including the grey literature [27, 28]. Publication rates may be misleading for women since they tend to have different publication strategies [29]; because of these strategic differences, some have called for metrics emphasizing quality and impact over quantity [26, 30].

The effects of these complex dynamics are especially profound in American archaeology, where the vast majority of practicing archaeologists are no longer academics or museum professionals (page 7 in [31]). In academia, researchers must “publish or perish”, and much time and energy are devoted to generating peer-reviewed publications. But, most archaeologists are now employed in the private sector in cultural resource management (CRM) firms or by state and federal agencies, organizations with fewer clear rewards (and less time and fewer resources allocated) for publishing in peer-reviewed journals. Professionals outside of academia, however, *do* produce a great deal of written output—output that is typically published in CRM and agency-produced technical reports and classified as “grey literature”. This volume of work, while significant, is a challenge to systematically track; despite increasing availability of these reports and citations in digital archives such as the Digital Archaeological Record (TDAR http://core.tdar.org/), there is no single national clearinghouse, and submissions to these archives are self-reported and biased because not all individuals or groups (agencies, private firms, etc.) systematically submit reports or citations. There are, however, some sources (e.g., un-refereed journals) that are widely available and published on a regular basis that can represent a wider extra-academic authorship. These publications, while relatively rare, offer a unique window into publishing trajectories that are typically passed over by productivity studies. In this study we tracked gendered publication patterns in one such journal, the Society for California Archaeology (SCA) *Proceedings*, and compared our findings with results from two peer-reviewed journals, the *Journal of California Anthropology*/ *Journal of California and Great Basin Anthropology* (*JCGBA*) and *California Archaeology* (*CA*). We also compared the occupational affiliation of authors who published in these venues, and found that more women and men working outside of academia publish in the non-refereed *Proceedings*.

We argue that this “peer review gap” can be best understood in terms of the structural realities of our discipline, including the fact that 9 out of 10 archaeologists work in places where publication in peer-reviewed journals is not explicitly rewarded or supported, and that women (in archaeology and throughout the sciences) continue to lag in the type of appointments that offer institutional support and where the academic publishing model is followed [7, 9, 16, 25, 32–36]. In terms of mechanics, we recognize that there are concrete differences in the costs and benefits of publication for archaeologists who are employed in range of roles, including cultural resource management and agency professionals, tenure-track faculty, and adjunct faculty. For example, while publication output is an important factor in the decision to grant tenure and promotions for professors, the benefits of publication for those in private and agency sectors is less clear. In other words, the benefits derived from generating peer-reviewed publications may not outweigh the often-substantial time costs for professionals outside of academia. Such differences may ultimately influence the decisions of people of all genders and backgrounds, but, because of the current structure of our discipline and societal roles/expectations that differentially influence the decisions and lived experiences of various genders, women and minority groups are overwhelmingly affected by these variables.

## Framework

Archaeology, among many other fields, has seen a major growth in the private sector and in agency work since the mid-1970s. Prior to this time, most archaeologists in the USA worked for universities and museums, like the famous archaeologist character Indiana Jones depicted in Hollywood films. Profound changes in the profession occurred after several key developments, including the passing of the Archaeological and Historic Preservation Act (AHPA), also known as the Archaeological Recovery Act, and the Moss-Bennett Bill, in 1974, which clarified the authority of Federal agencies to fund archaeological investigations designed to mitigate potential impacts of their activities and project developments on archaeological resources. Importantly, the AHPA put pressure on land managers to comply with archaeological responsibilities as stipulated in earlier key legislation, including the National Historic Preservation Act (NHPA) of 1966 and the National Environmental Policy Act of 1969 [37, 38]. Another key development was the incorporation of the concept of National Register of Historic Places (NRHP) eligibility into Section 106 NHPA regulations, also in 1974, which provided a specific mechanism for the evaluation of archaeological sites. Consequently, there was a sudden expansion of “compliance archaeology”, an umbrella term referring to Section 106 and related management activities conducted by federal and state agencies, as well as private sector cultural resource management (CRM) firms (an industry that employs archaeologists as well as other heritage management professionals, such as historians, architectural historians, and ethnographers). In sum, the modern landscape of professional archaeology is profoundly different from what it was 50 years ago; approximately 90% of archaeology is completed in CRM contexts (page 7 in [31]), and compliance work accounts for an estimated $1 billion dollars of annual spending by public agencies and private sector companies (Page 297 in [39].

### Role of publication in different employment sectors

In terms of publishing, these historic changes are relevant because the vast majority of archaeologists today are employed in positions that do not follow an academic publishing model. Namely, while peer-review publication is a highly rewarded and generally supported activity in academia, rewards for peer-reviewed publishing are typically less clear and pronounced in compliance archaeology, and compliance positions do not allocate much (or any) time for publishing scholarly papers that are not work-related requirements. Compliance archaeologists do, however, produce a substantial corpus of professional output, but, by and large, the vast majority of the information generated by these activities is reported in technical reports, often referred to as the “grey literature.”

The emergent professional landscape of American archaeology has thus produced two “parallel literatures”, simplified here as: (1) ***Peer-reviewed/ academic model publications*:** Journals, books, and monographs that follow an academic model of publishing, and include international, national, and local venues that are peer-reviewed or refereed by one or more experts in the field; (2) ***Professional*, *industry*, *and “grey” publications*:** Publication venues that include a wide range of written sources often referred to as the “grey literature”, and, within archaeology, primarily consist of technical reports produced largely by private firms and agencies (often internally and/or externally peer-reviewed), authored pieces in un-refereed journals, and society newsletters.

Archaeologists have debated the quality and place of these literatures in the production and dissemination of knowledge (e.g., [40–43]). Peer-reviewed publications are generally more prestigious than the grey literature because of the assumed higher quality associated with the peer-review process, which is designed to weed out poor-quality research. In reality, there is a wide range of quality in both publication categories, though there is a persistent and often misguided tendency to conflate grey literature with poor quality and with entirely un-refereed works. The upshot is that although this body of work contains material representative of a wider breadth of participants in the field of archaeology [27, 28], it is overlooked, and academics often perceive publication in these venues as less prestigious and less likely to be rewarded.

One of the difficulties of using publication rate to evaluate professional productivity in archaeology is that there is significant disparity between the academic publishing model and the reality of modern archaeology, namely that most archaeologists are employed where publication in peer-reviewed journals is typically not explicitly rewarded or supported. These dynamics underlie the overall structure of our discipline, and they are especially relevant when evaluating gendered publication patterns: While there is overall gender parity in terms of the demographic representation of women and men as participants in the field and in degrees awarded, women continue to lag in academic appointments (especially in tenure-track positions at high-tier R1 institutions) [9, 16, 25, 32–34, 36, 44–45] where publication output is important and people have institutional support and time allotted for writing.

This incongruity between the lived experiences of professionals within and outside of academia warrants a reevaluation of the criteria that we use to conceive of and assess the output of practitioners in archaeology and in other disciplines. In an effort to assess the impacts of gender and institutional affiliation on professional productivity beyond the standard metrics that have traditionally been used to evaluate equity in publication rates, we employed a method that tracked the two general groups of publications with the methods described below.

## Materials and methods

This case study speaks to archaeological publication trajectories of journals that cover a large area of western North America (California and Great Basin cultural areas, as below). Archaeologists working in this region account for a substantial number of professionals working in the USA. For example, more members of the Society for American Archaeology reside in California than any other state (as of 2015, 311 or 14.7% of 2120 members in the USA); the same is true for the Register of Professional Archaeologists (as of 2017, California accounts for 451 or 17.6% of the 2556 members in the USA). Furthermore, in both societies, archaeologists from California and Great Basin states account for over a third of all USA members (Table A in S1 File). Data gathered from the Member Directory of the American Cultural Resources Association indicates that CRM firms from these states account for nearly one fifth of all CRM firms and organizations in the USA; California has the second highest number of CRM firms/organizations next to Pennsylvania (Table A in S1 File). In addition to CRM, because state and federally owned lands in the western states are abundant, numerous archaeologists in the region work for government agencies (the Bureau of Land Management, the US Forest Service, the National Park Service, etc.). Finally, there are numerous post-graduate programs in archaeology regionally, especially in California, which has the highest number of PhD programs with an archaeology focus compared to any other state except for New York (n = 6; or 8% of 75 programs in the USA) (Table A in S1 File).

### Baseline participation

Quantifying the number of female practicing archaeologists in the California and Great Basin study area was a challenge. The best place to obtain these data are from the major regional society, the Society for California Archaeology (SCA), which is the largest state archaeology society in the USA, with a membership that currently approximates 1100 individuals, and annual meetings that regularly attract upwards of 800 people [46]. Notably, the Society’s reach extends beyone the state of California; According to the SCA’s mission statement, the organization is fundamentally committed to the “California and the regions that surround and pertain to it,” and many members include CRM employees and academics that work throughout the western States. While the SCA does not keep statistics on the gender of its members, annual meeting participants, or annual meeting paper presenters (Denise Wills, personal communication), we were able to estimate women’s participation in the society by calculating the gender of members recorded in newsletter mailing lists supplied to us by the SCA. We recorded the gender of individuals (male, female, or unknown) for all mailings sent during six one-year periods that were selected between 1967–2011; we spaced these data years after 1971 in roughly 10 year intervals, but 2001 data were not available.

### Journal data and background

We tracked longitudinal publishing trends of men versus women by considering the three major venues for publication of California and Great Basin archaeological research, including two peer-reviewed journals: the *Journal of California and Great Basin Anthropology/ Journal of California Archaeology* (*JCGBA/ JCA)* and *California Archaeology* (*CA*), and the non-peer-reviewed Society for California Archaeology *Proceedings* (a grey literature publication, as noted above). All three publications vary in purpose, content, and audience (see Journal summaries, Text A in S1 File). *CA* and *Proceedings* publish articles mostly on the archaeology of the California culture area, which includes the state of California and southwest Oregon (“Alta California”), as well as Baja, Mexico (“Baja California”). The longest running journal, *JCGBA/JCA*, has the greatest geographic breadth, and publishes articles on California (again, both Alta and Baja California), and the Great Basin culture area, which includes parts of eastern California, southeast Oregon, Nevada, Utah, western Colorado, northern Arizona, southern Idaho, western Wyoming).

### Publishing output

We determined the longitudinal publishing trends of men versus women by tracking gender and occupational data for all authors for the entire publication history (up to the year 2016) of the three journals used in this study. The longest running journal is *JCGBA/JCA*, established in 1974; *Proceedings* was first published in 1984, followed by *CA* in 2006 (Text A in S1 File). We limited our sample to peer-reviewed articles and reports in *CA* and *JCGBA/JCA*, and excluded editor’s notes, book reports, news and notes, and other similar contributions that are largely non-refereed. The *Proceedings* sample included all contributed papers, but not editorial notes or front matter. For each article sampled, the year, the total number of authors identified as men versus women, the total number of men versus women lead (first or sole) authors, and the occupational affiliation of lead authors (see below), was tabulated.

### Gender

In the above data set, we designated individuals as “male,” “female,” or “other.” (Note that we are speaking of social, not biological, categories, and in this article we use the terms “females” and “women” interchangeably, as we also do with “males” and “men”, which is the convention in much of the published literature on gender equity issues in publishing). In the case of ambiguous names for authors who we did not know personally, we were often able to discern gender from a simple internet search. For example, biographies of many authors are readily accessible on departmental, professional, or social media websites, and the gender of the author could be discerned through gender-specific pronouns used in the text and supported through the presentation of gender through photographs. When this method was not successful, we asked past and present editors of the journals and other colleagues for assistance. When the gender of an author could not be discerned, was ambiguous, or was queer/genderqueer/non-binary, we designated the author as “unknown.” A limitation of this study is that we were unable to fully assess the representation of queer/genderqueer/non-binary individuals due to our methods, which did not include self-reporting.

### Occupational affiliation

To better understand occupational patterns of journal contributors over time, we tracked the occupational affiliation (stated with journal article bylines) of all lead authors. We divided affiliation into seven general categories: (1) ***Academic***: Mostly universities and colleges, but also academic museums, and academic CRM firms; (2) ***Private sector/ CRM firms*:** Cultural resource management firms and entities; (3) ***Agency***: Federal, state, and local agencies; (4) ***Museums***: Public or private nonprofit museum institutions; (5) ***Independent***: No affiliation stated or only an address; (6) ***Other*:** Any other affiliation not clearly associated with the above categories, including historical societies, research groups, international research institutes, and Tribal organizations (including both authors with tribal membership and authors who work for Tribal organizations; listed as a separate category in **Tables I-K, SI**); Ten lead authors in *Proceedings* and three authors in *JCGBA/ JCA* who listed more than one affiliation were also included in this category. Note that these affiliation categories cannot take into account the various stages of each author’s career (e.g., “academics” is a catchall that includes students as well as professors and teachers at various institutions).

## Results

### Baseline participation

**Fig 2:**
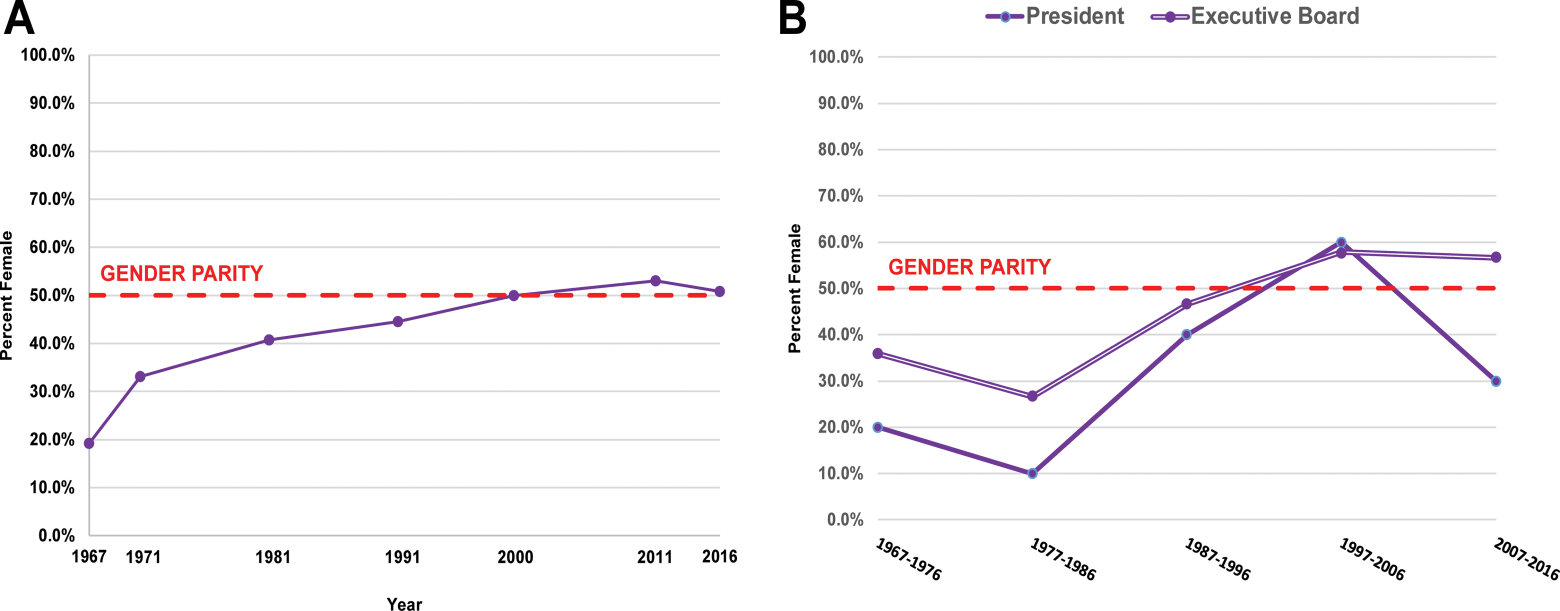
(A) Gender composition of SCA members, select years between 1967–2016. (B) Gender composition of SCA President and Executive Board members (including 49 Presidents, 40 Northern Vice Presidents, 41 Southern Vice Presidents, 42 Secretaries, and 30 Treasurers), 1967–2016, from Origer and Gilreath (page 241 in [47]).

Baseline participation of women in our regional sample of SCA archaeologists has steadily risen over the past four decades and today equals or exceeds that of men according to several metrics. Although the SCA membership was overwhelmingly dominated by men in its first year (80.8% men in 1967), the number of female SCA members steadily rose during the following years (Fig 2A; Table B in S1 File). By 2000, roughly half of the SCA members were women, and by 2011 their numbers surpassed men. A similar trend is apparent in the SCA’s leadership. Between 1967–2016 a total of 202 individuals served on the SCA Executive Board, and there is an overall increase in women members (Fig 2B). Origer and Gilreath summed up the situation: “The earliest decades of the SCA reflect a preponderance of males on the Executive Board. However, females often held the positions of Secretary and Treasurer throughout all decades. The 1997–2006 decade is unique in that it is the first time females outnumbered males in three of the five positions, and when the president was more often female than male” (page 240 in [47]). Notably, the SCA Women in California Archaeology Committee (WCAC) was formed in 2012, and is actively addressing a range of gender parity and sexual harassment issues; task forces include several specifically engaged with increasing nominations of women for SCA Awards and leadership roles, as well as increasing publications and mentoring opportunities [48].

### Membership category and age

**Fig 3:**
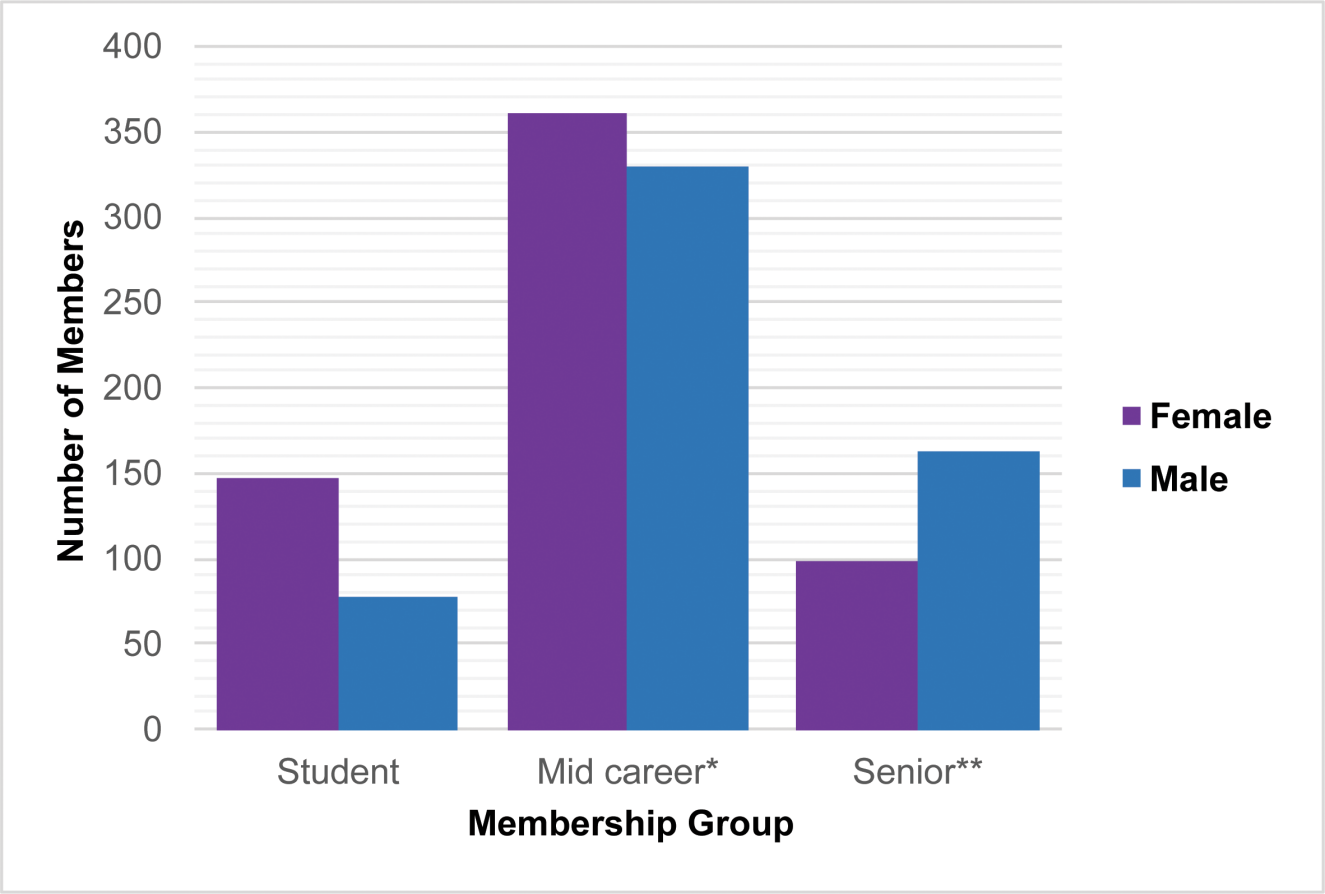
Gender composition of general age/ career stage categories of SCA members, 2016. *Mid-career (Includes regular, contributing, lifetime, spouse, and admin members); **Senior (Includes senior, senior lifetime, and contributing senior members) (Unknowns removed).

We also examined the gender representation of different membership categories and general age groups for two recent years, 2011 and 2016. First, we looked more closely at the composition of different membership categories (Table C in S1 File). Women slightly surpass men in overall membership in both years, but more women are designated “spousal” members (78.3% in 2011 and 66.7% in 2016), and, in terms of general age trends, women outrank men in younger membership categories. In 2016, for example, women accounted for 63% of students (who tend to be the youngest members and/or are at the earliest stage of their career) and 52% of “mid-career” memberships, but only 38% of senior memberships (Fig 3).

### Annual meeting participation

**Fig 4:**
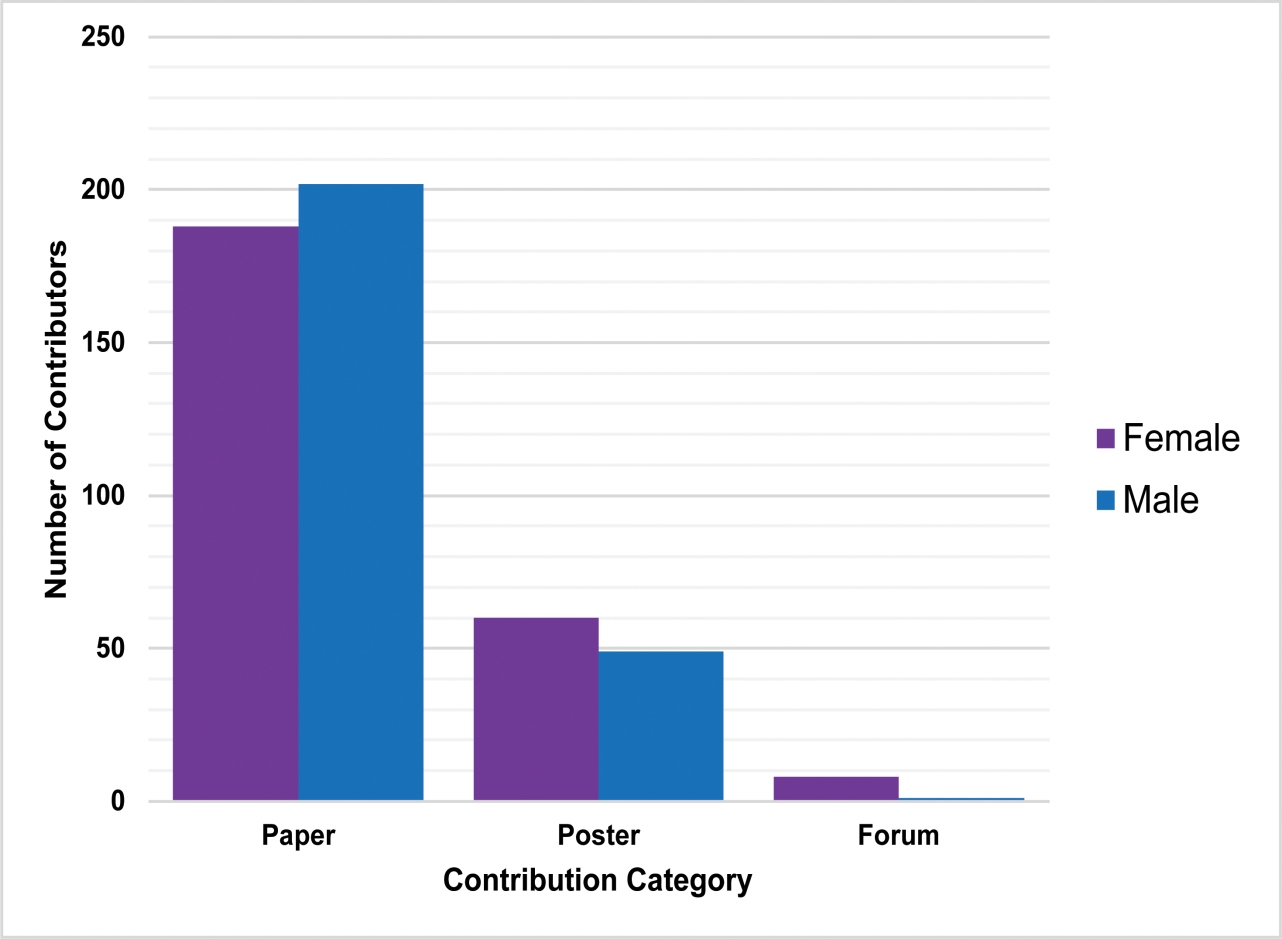
Gender composition of SCA 2016 Annual Meeting Contributors by contribution type (Note: 4 unknowns removed; retained in Table D in S1 File).

Next, we looked at the gender composition of contributing participants for two recent years of the SCA Annual Meeting—the 2011 meeting held in Rohnert Park, and the 2016 meeting held in Ontario, California. By compiling data recorded in the annual meeting program, we found that women are substantial contributors at meetings: 126 women and 151 men were authors in the listed papers, posters, and forums (Table D in S1 File). In both years, women participated in more poster sessions than men, while men presented more papers (Fig 4). There is a statistically significant relationship between gender (unknowns removed) and contribution category for 2011 (χ^2^ *= 11*.*9984*, *p =* .*002481*. *<* .*01*.) and 2016 (χ^2^ *= 7*.*026*, *p =* .*029807*. *<* .*05*). Though not the focus of our paper, more longitudinal data are needed to better understand these dynamics.

**Fig 5:**
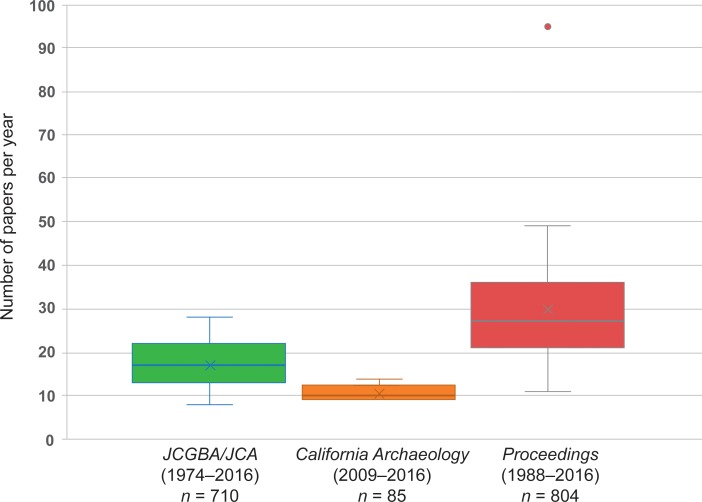
Box and whisker plot of number of papers per year in peer-reviewed and non-peer-reviewed journals.

In sum, our findings reflect national data indicating that women are major participants in the field of archaeology (Fig 1), [5]. In the SCAs earliest years, there were far more male members than females, but today women equal or exceed men in terms of number of participants. Women are also major contributors at the SCA annual meetings, and, at least in recent years, they tend to present more posters and fewer papers than men. If the large number of student participants who are women is any indication of future participation trends, it seems likely that an increasing number of women will participate in SCA meetings in the coming years. But how are women faring in terms of publishing? This is a question we turn to in the next section.

### Publishing trends

We examined 1,599 papers found in the three publications included in the study (Fig 5; Table E in S1 File). In terms of number of papers published per year, the *Proceedings* is by far the most variable of the publications considered in this study, with a low of 11 papers in 2002 to a high of 95 in 2009 (in 2008 no papers were published). This variability is a result of the lack of page limits in the *Proceedings*: it publishes all submissions as long as they were presented at the previous year’s SCA annual meeting and meet the SCA Bylaws and Ethical Guidelines (Text 1 in S1 File). In contrast, *JCA/ JCBA* and *CA* have space limitations. Between 1974 and 2016, *JCA/ JCGBA* has published an average of 16.9 papers per year/ volume, although the number of papers has declined over the years as the page length of papers has increased over time. *CA* publishes the fewest number of articles per year, averaging 10.6 papers per year/ volume between 2009 and 2016.

### Gender trends

For the three journals we examined, we found that between 1974 and 2016, 2,617 authors had papers published. Of those authors, 844 (32.3%) were women, 1,762 (67.3%) were men, and 11 (0.4%) were gender unknown/ambiguous. After tabulating these data, we calculated the percentage of women authors in every journal issue as a way to compare historic gender participation rates. While we demonstrated that women’s participation in the non-refereed source (*Proceedings*) has been historically robust and remains strong today, we found a profound and persistent gender gap in the two peer-reviewed publications, *JCA/JCGBA* and *California Archaeology*.

**Fig 6:**
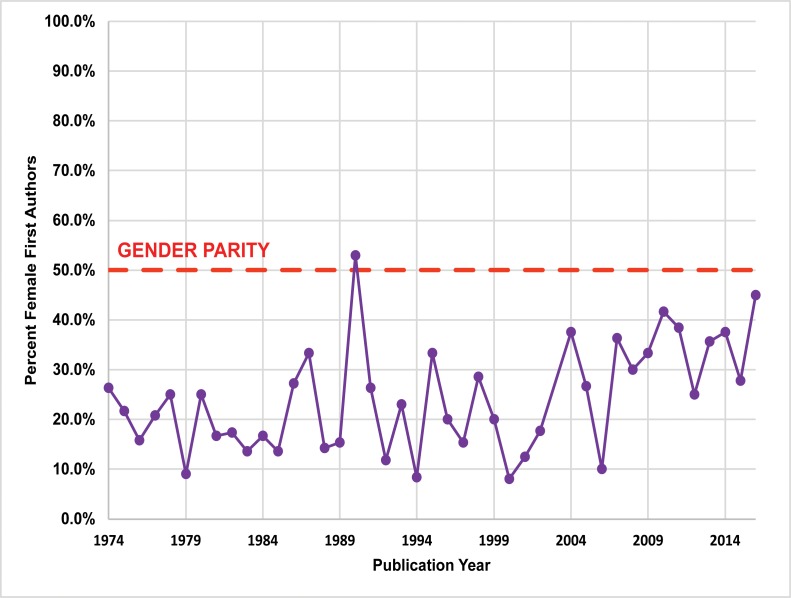
Publication rates for female lead authors published in the *Journal of California Anthropology* (*JCA*)/ *Journal of California and Great Basin Anthropology* (JCGBA) (1974–2012). Note: the journal was not published in 2003. The proportion of female lead authors significantly increased between 1974 and 2016 (*P* < .01, by the Cochran-Armitage χ^2^ test for trend).

**Fig 7:**
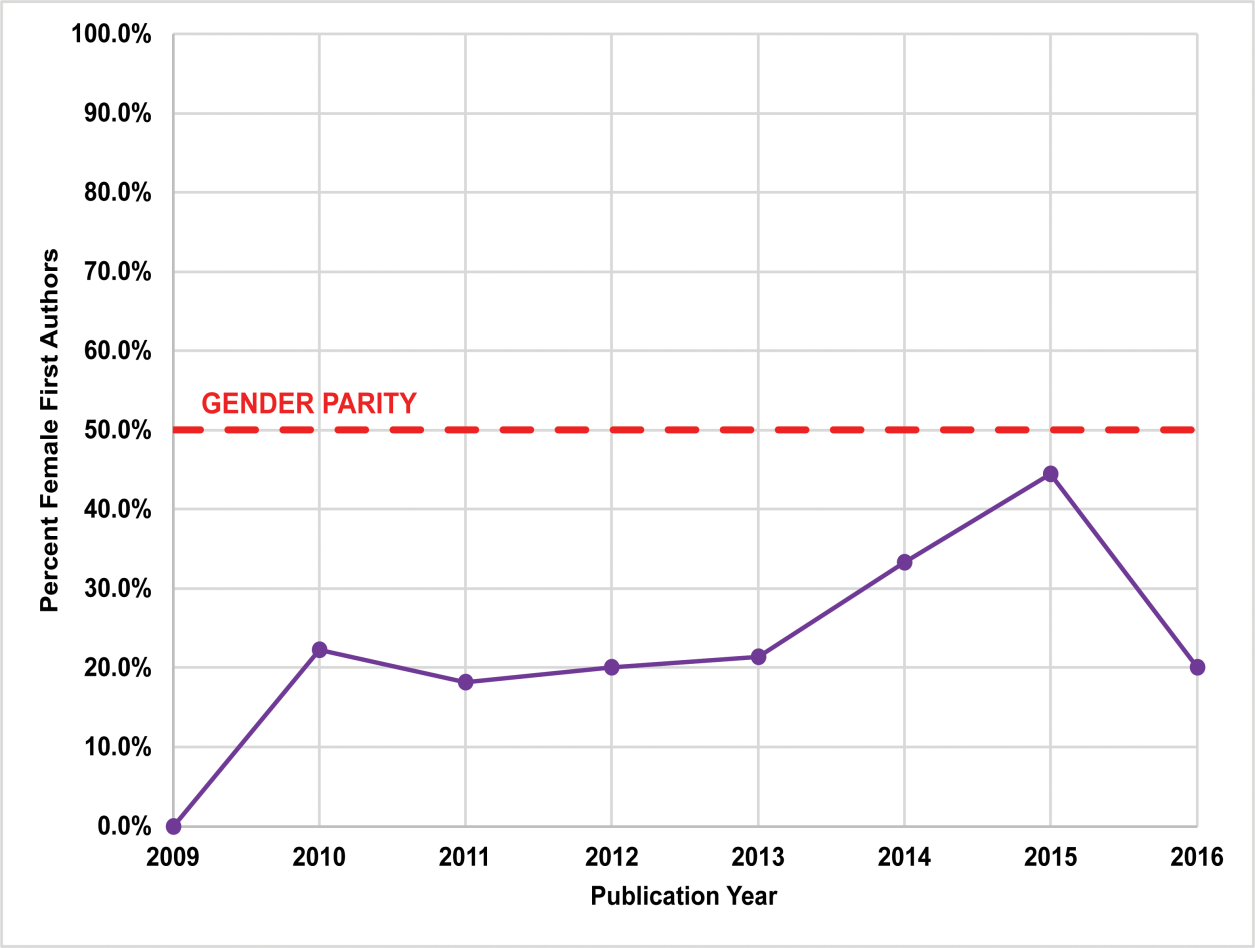
Publication rates for female lead authors published in *California Archaeology* (2009–2016).

**Fig 8:**
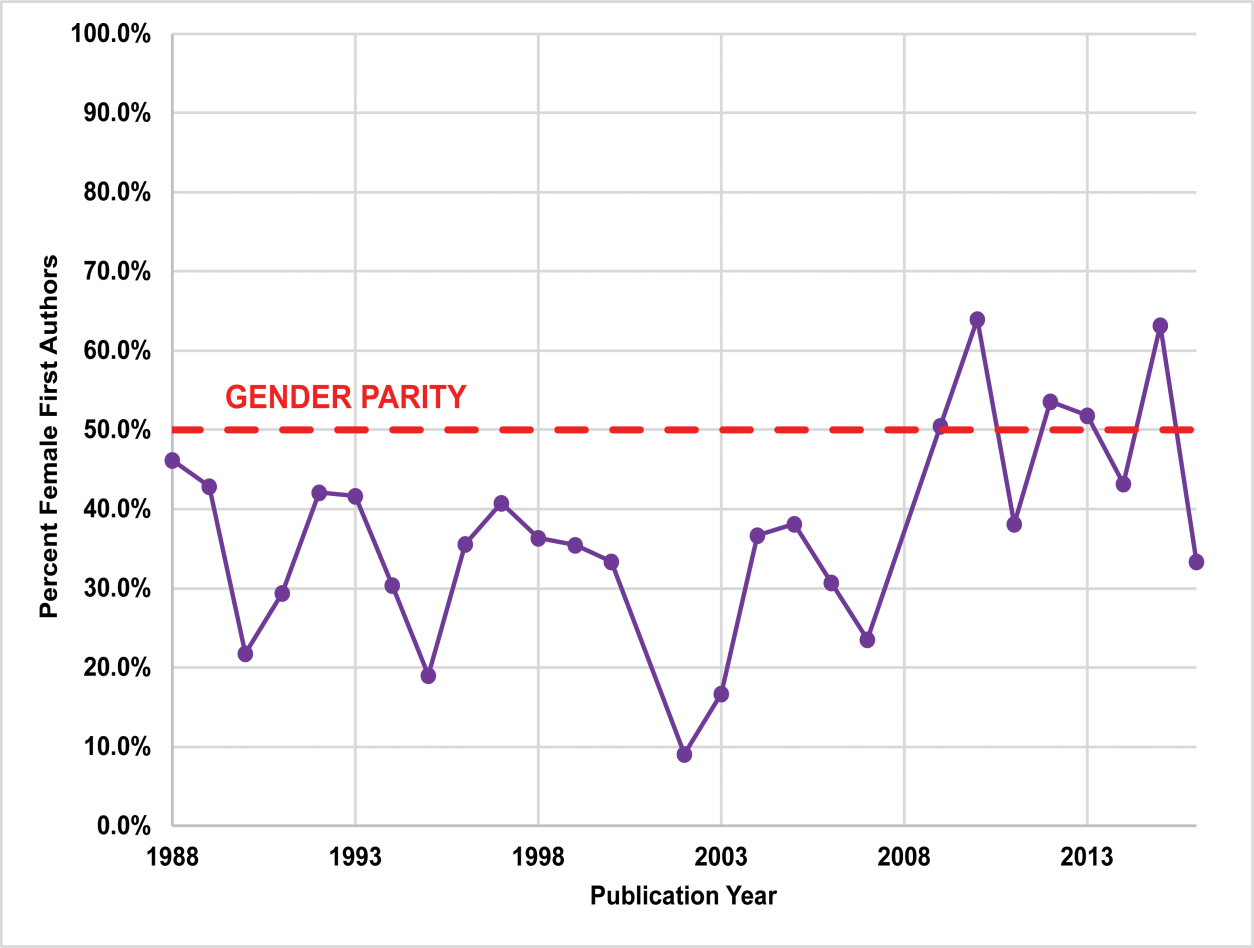
Publication rates for female lead authors published in SCA *Proceedings* (1988–2016). The proportion of female lead authors significantly increased between 1988 and 2016 (*P* < .01, by the Cochran-Armitage χ^2^ test for trend).

Between 1974 and 2016, women’s participation in *JCA/ JCGBA* (1974–2012) is comparatively low. Of the 710 papers examined, 166 (23.4%) had female lead (first or sole) authors, and 294 (24.9%) total female authors (Fig 6; Table F in S1 File). Women published the least in the region’s newest publication, *California Archaeology*, with 18 (21.2%) female lead authors and 69 (33.8%) total female authors between 2009 and 2012 (Fig 7; Table G in S1 File). In contrast, the SCA *Proceedings* overall had the highest percentage of female authors (Fig 8; Table H in S1 File). Between 1988 and 2015, 804 papers were published. Of these papers, 313 (38.9%) had female lead authors, and 481 (39.0%) had female contributing authors.

### Longitudinal trends

We performed statistical analysis on our publication data using the Cochran-Armitage χ^2^ test for trend [49, 50]. While there is substantial year-to-year variation in gendered patterns of authorship (Figs 6–8), our results indicate significant longitudinal increases in female lead authors for the two longest running journals (*P* < .01): *JCGBA/ JCA* (*P*-value = 0.0054 < .01) and *Proceedings* (*P*-value = 0.0062 < .01) (Figs 6 and 8). A significant proportional trend was not indicated for *CA* (*P*-value = 0.0652 > .01), but this journal has only been published for a relatively short period (seven years) (Fig 7). Notably, female authorship in *Proceedings* is particularly robust after 2009, the year the journal went online: female lead author proportions are close to or exceed parity (50%) after 2009.

### Occupational affiliation

**Fig 9:**
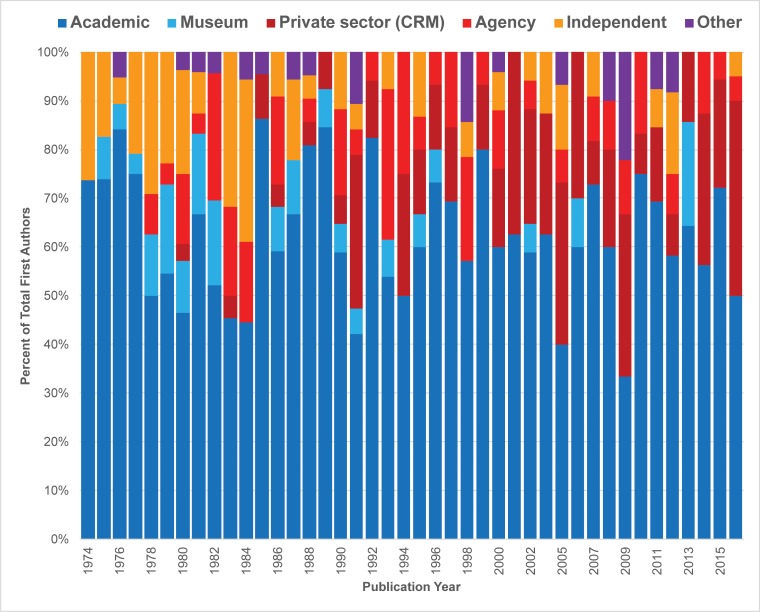
Percent distribution of lead author affiliations, *JCGBA/ JCA* (1974–2016).

**Fig 10:**
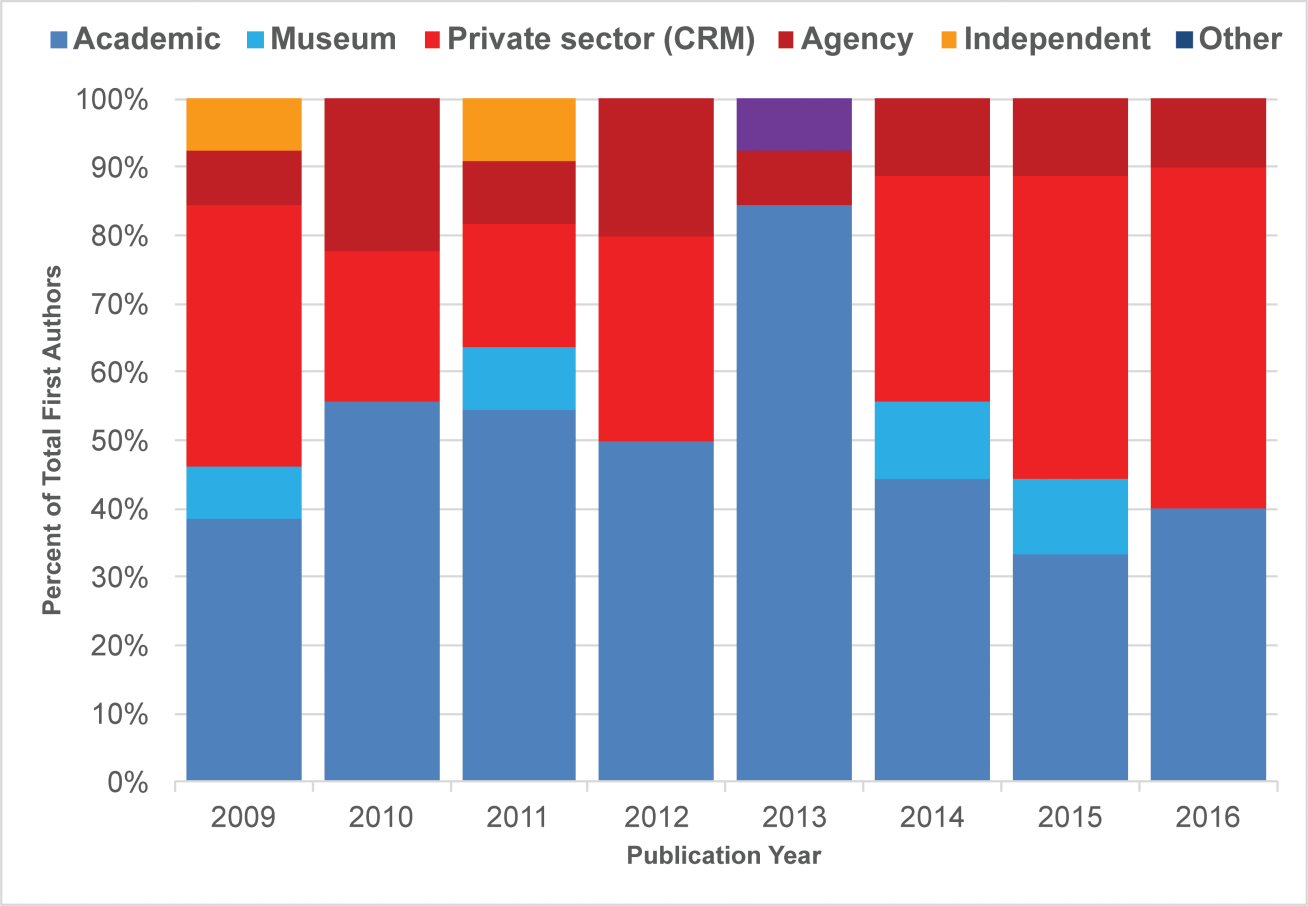
Percent distribution of lead author affiliations, *CA* (2009–2016).

**Fig 11:**
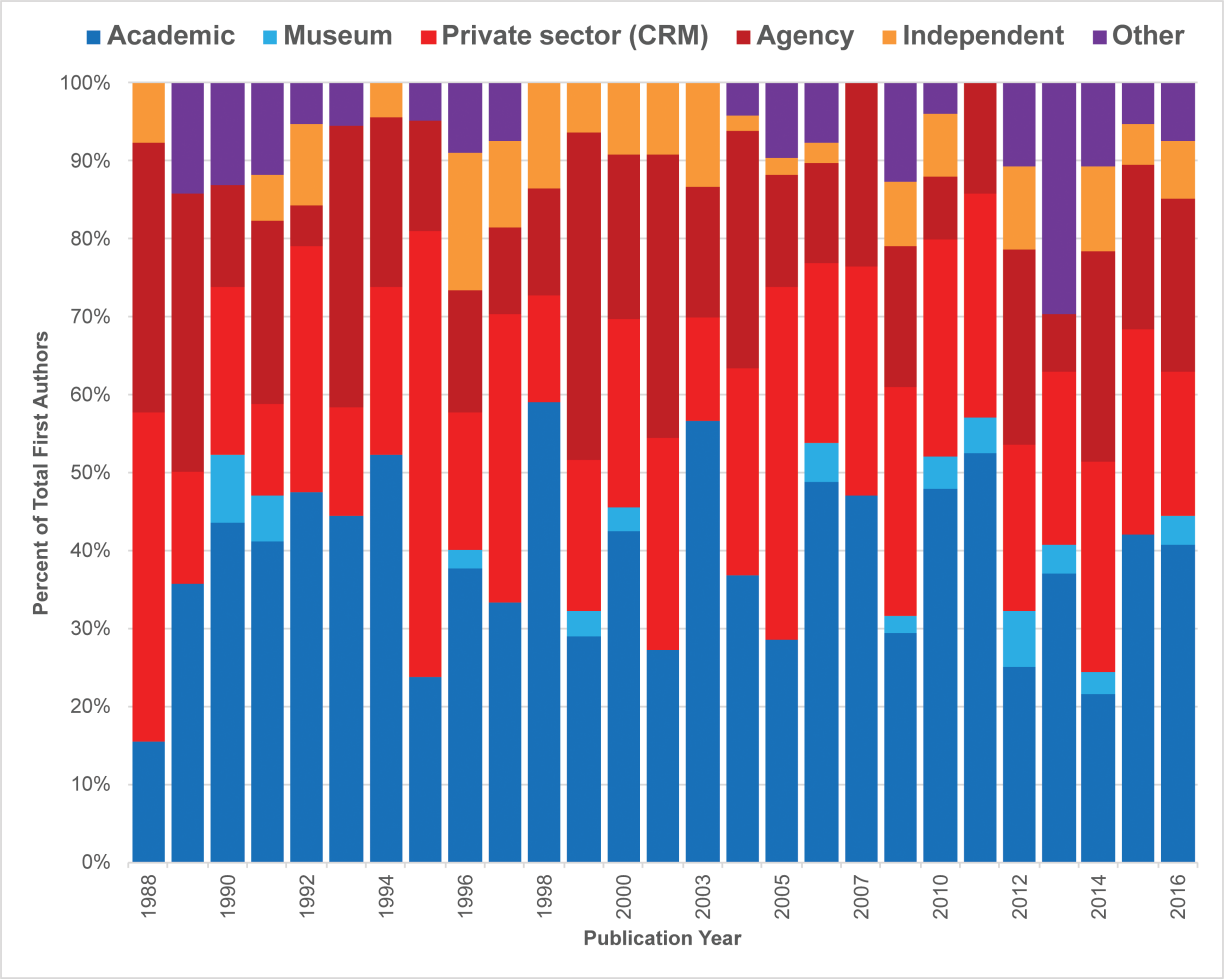
Percent distribution of lead author affiliations, SCA *Proceedings* (1988–2016).

We also noted longitudinal and gender trends in our analysis of occupational affiliation of lead authors (Figs 9–11). The overall shifting occupational landscape of professional archaeology is most apparent in the longest (42-year) record of publications we tracked, *JCGBA/ JCA*. In the early 1970s, most first or single authors were affiliated with academic institutions or museums (shaded blue and light blue, Fig 9). This reflects the general trajectory of careers in archaeology as a discipline during that time period. However, career opportunities began to shift in the late 1970s, as archaeology evolved into a discipline dominated by compliance (concerns overseen by private sector firms and federal and state agencies). While within *JCGBA/ JCA* most first authors tend to have academic credentials across time, a growing number of “compliance archaeologists” are making their authorial mark in the journal. This group of professionals (shaded red and dark red in Fig 9) began to publish as early as 1978 (agency authors) and 1980 (private CRM authors), and their contributions have consistently increased over time. *California Archaeology*, a journal that began publication in 2009, reflects the professional landscape of archaeology from that time to the present: agency and private sector/ CRM professionals contribute a great deal, in many years contributing as many or more papers than the academic and museum group (Fig 10). Overall, the non-peer-reviewed *Proceedings* demonstrates much greater diversity in lead author affiliation, and proportions of categories are much more variable compared to the peer-reviewed group (Fig 11). One of the most striking findings in this regard is the relatively low number of lead authors with academic affiliations publishing in the journal, which is likely associated with the perceived low merit of non-peer-reviewed publications in tenure reviewed academia. Museum affiliations are also relatively low in the *Proceedings*. However, many other archaeologists with other affiliations publish in the journal, especially those associated with agencies and private sector/ CRM firms, a trend that is consistent throughout the lifespan of the journal. Notably, the “other” category is more substantial and diverse in *Proceedings* in comparison to the peer-reviewed venues, and includes authors who do not publish or only publish rarely in the peer-reviewed journals, including 25 authors affiliated with the Instituto Nacional de Antropología e Historia (INAH), Centro Baja California, 11 authors affiliated with regional and topical societies, research groups and committees, 10 authors with more than one affiliation, two site stewards, and two authors affiliated with Tribal organizations.

#### Gender and occupational patterns

**Fig 12:**
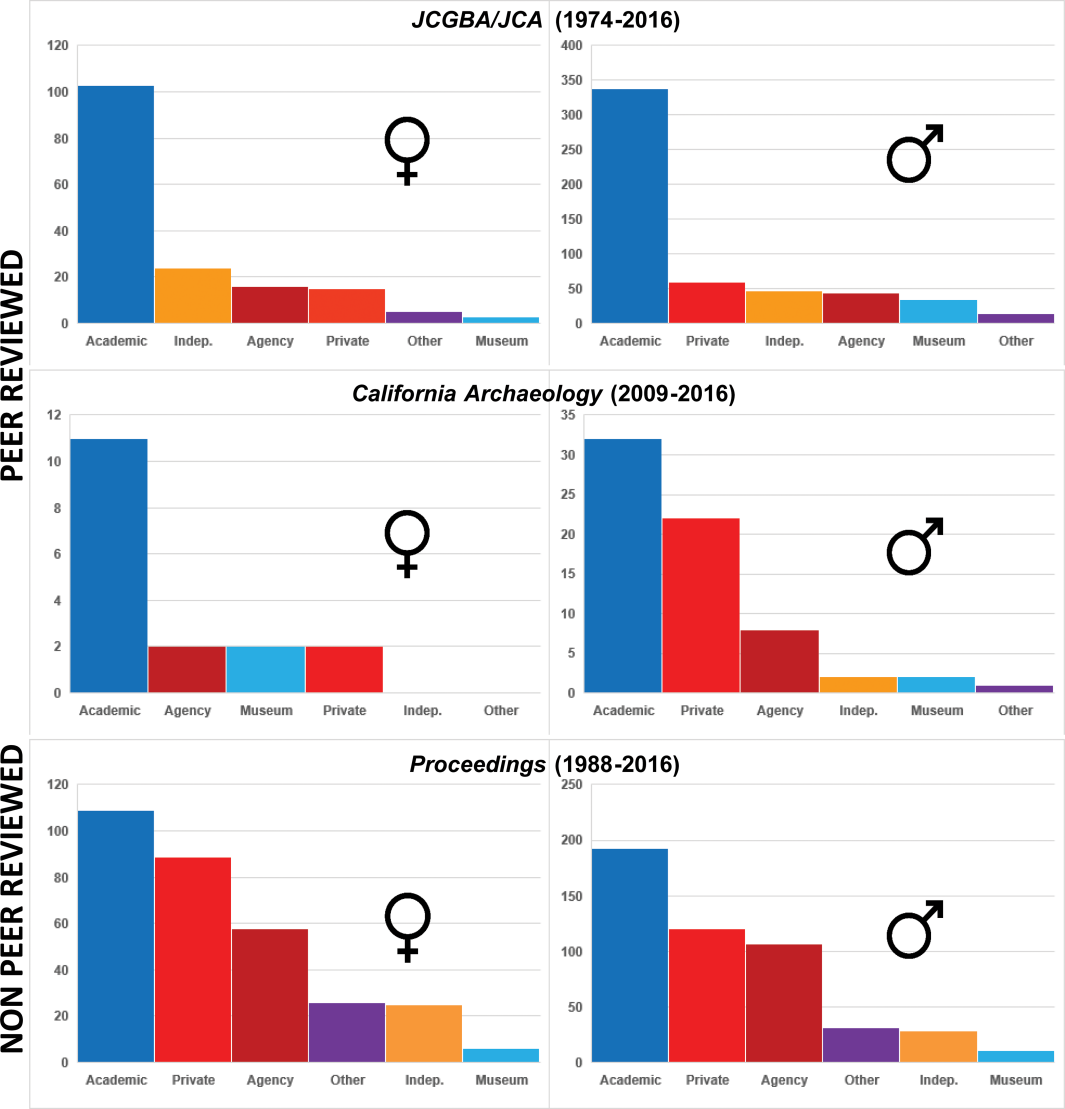
Occupational affiliation of female and male lead authors, in descending order of frequency. Top: *JCGBA/ JCA* (1974–2013), Middle: *California Archaeology* (2009–2016), Bottom: *Proceedings* (1988–2016).

Our comparison of the gender and occupational affiliation of lead authors revealed several interesting trends (Fig 12; Tables I-K in S1 File). Of the female lead authors we tracked who publish in peer-reviewed journals, the overwhelming majority were employed in academia (62% in *JCGBA*/ *JCA* and 64.7% in *CA*) (Fig 12; Tables I-J in S1 File). We observed a similar trend for men in *JCGBA*/ *JCA*, in which 63.0% of male lead authors are academics, followed by private sector/ CRM, independent, and agency authors, respectively (Fig 12; Table I in S1 File). However, a striking pattern emerged with *CA*, which is evidently a target journal for many male authors working in non-academic settings: although most authors are academics (47.8%), there are noticeably higher percentages of men associated with private sector/ CRM entities (32.8%) than are found in *JCGBA/JCA*. Conversely, there are markedly lower numbers of women with affiliations outside of academia in *CA*, and only 11.8% are private sector/CRM archaeologists (Table J in S1 File). Why women authors working in the private sector/ CRM and agencies are publishing so little in this journal is unclear. However, many more women (and men) from outside of academia are publishing in the non-refereed *Proceedings*. In *Proceedings*, the affiliations with the highest percentages of authors are, in order, academics (34.8% of female leads; 39.4% male), private sector/ CRM (28.4% female, 24.5% male), and agency authors (18.5% female; 21.8% male). Clearly, the percentages of extra-academic lead authors are significantly higher in *Proceedings* than in *JCGBA*/ *JCA* for both women and men, and are also higher than those for women in *CA* (Fig 12; Table K in S1 File). Notably, the roughly equal proportions of male and female authors in each affiliation category in *Proceedings* suggests that there is greater equity in this journal’s publishing patterns.

## Discussion

Our analysis of publication data from three journals indicates that women and extra-academic professionals are the least likely groups to publish in peer reviewed journals. A notable exception to this patterns involves male compliance archeologists who are publishing extensively in the peer-reviewed *CA*. Due to the relative infancy of this journal, however, the sample size is small, so the future evolution of this exception will be interesting to track. We attribute current accessibility trends to a complex suite of factors that include, but are not limited to, the overall structure of our discipline, gender socialization and cultural norms, and variation in the time costs and benefits associated with different publication categories (see Framework section, above). Our analysis is especially revealing of the disciplinary structure and occupational cost-benefit realities of different occupational settings within the field of archaeology.

### The role of publication and disciplinary structure in American archaeology

For academics, there are many clear benefits to investing significant time and effort into publishing in peer-reviewed journals. Tenure, especially in R1 institutions, depends on an individual’s success in publishing, as well as on grant writing, teaching, and service. Publication output, especially in peer-reviewed journals, is a highly valued and highly rewarded metric, and the system is designed to reward scholars with high output with tenure and coveted faculty positions. Likewise, a lack of productivity is punished directly; academics with few or no publications are less competitive for post-doctoral and tenue-track positions, have lower grant success [51] and lower retention rates [52], and receive fewer promotions [29, 52]. Therefore, publication in peer-reviewed journals is a measure of relative productivity and level of influence in academia, is critical to promotion and tenure, increases social capital, and confers a level of intellectual legitimacy [13, 14].

For the majority (90%) of American archaeologists who work in compliance archaeology (page 7 in [31]), there is an emphasis on the production of professional technical reports and similar written deliverables, but the benefits of academic model publication are much less clear. Although peer reviewed publication might be advantageous, it is often not a requirement for success in these professions. Furthermore, sufficient time is typically not allocated for this type of writing, and in many cases, there are no clear financial or promotional rewards for individuals with higher publication rates than their professional counterparts.

While there is overall gender parity in archaeology terms of the demographic representation of women and men as participants in the field and in degrees awarded, women continue to lag in higher tier and more prestigious positions within the profession. Within the compliance industry, there is some evidence that men more commonly occupy senior leadership positions [46]. This disparity is very clear in academic (especially high-tier R1 tenure-track) positions [9, 16, 25, 32–34, 36, 44–45] where publication output is important and there is institutional support and time allotted for writing. For academics in adjunct and temporary positions, publication is an added activity, which can make transitioning to tenure-track difficult due to lack of time for the very activities that would allow access to coveted positions.

### Bridging the peer-review gap: *Proceedings* as a multi-vocal model for the dissemination of knowledge

Dissemination of results remains a major challenge in archaeology today [53–54]: “Many of the technically best and most substantively informative archaeological studies currently being done in North America are those done in a CRM context. Yet, the most significant results of much of this work often remain effectively hidden from other archaeological researchers, let alone from the general public” (page 50 in [54]). The current structure of our discipline simply makes it a challenge for non-academics to allocate the time required for ferrying an article through the peer review process, which is typically very time consuming and, for these professionals, a largely unrewarded and unsupported activity. Publication under these circumstances often relies on individual initiative [54], but clearly this is not enough. A number of solutions have been fielded, including allocating a percentage of field project funding to synthetic publications, and/or major projects to produce peer reviewed papers so ideas can be funneled into “long established scholarly publication systems” (page 51 in [54]). Until such structural changes occur, however, non-refereed publications may provide an alternative means of bridging the peer-review gap.

Journals such as *Proceedings* offer publication opportunities for professionals who are not willing or able to pay the time costs associated with publication in peer-reviewed journals. As documented here, this seems to be especially true for women and people working in extra-academic settings. The process for publishing in *Proceedings* is straightforward and open to any SCA member who presented a paper at the previous year’s SCA Annual meeting (Text A in S1 File). Thus, the *Proceedings* provides an accessible means of disseminating research in a timely and relatively low cost manner (reduced time investment) for those who do not have the time (or desire) to publish in peer-reviewed journals. An added benefit of the journal is that it follows an “open access model” with no associated publication fees for authors; there are no space limitations for papers and, since 2009, all content is freely available to the public at no cost. In contrast, the peer-reviewed *CA* is behind a firewall and only accessible to subscribing SCA members and paying readers, and recent issues of *JCGBA/ JCA* are not accessible online (although, as of this writing, digital versions of issues prior to 2014 are now freely available online).

Our results indicate that women and CRM and agency professionals are taking advantage of the opportunity the *Proceedings* provides. For those working in compliance archaeology, the journal can be an important avenue for disseminating major findings from technical reports that might otherwise be overlooked. An added benefit is that it promotes “multi-vocality”, which Seymour points out is one of the significant advantages of the grey literature [27]. While our study only tracked gender and occupational patterns, we think venues such as the *Proceedings* may also help to make publishing more accessible to underrepresented groups, such as People of Color, who constitute less than 20% of American students in archaeology graduate programs [55]. This is important because People of Color, (who are often the subject of archaeological studies in North American Archaeology, for instance First Nations and American Indian people), are vastly underrepresented among full-time faculty members, are more likely to be employed at non-tier 1 universities and colleges, and experience biases in hiring and promotion [56–57]. Similarly, non-heteronormative and non-cisgender individuals are poorly represented and report experiencing harassment and discrimination in academic environments [58–59].

In archaeology, because most grey literature consists of technical reports that are often difficult to access, venues like *Proceedings* can provide a means for professionals from diverse backgrounds to disseminate their findings. While the overall impact of these publications is hard to predict, even if the potential readership is small, we think it is a benefit to the discipline to democratize the dissemination of ideas. This is not to say that non-peer reviewed journals are a replacement for peer-reviewed publications. Indeed, our aim should remain to close the peer-review gap, but until we solve some of the structural problems with dissemination outlined above, non-refereed venues may be the only realistic option for many individuals at present.

## Conclusions

This paper tracked the research output of archaeologists comparing articles published in two peer-reviewed publications (the traditional measuring stick of scientific productivity) with those in a non-peer-reviewed venue (representing a critical but largely overlooked corpus of work in archaeology) from western North America. Women are now well represented in the field, particularly among students and professionals in the middle stages of their career, but remain poorly represented in peer-reviewed publishing venues. Publishing trends suggest that the peer-review process acts as a major barrier to women publishing in archaeology journals, despite high levels of participation in terms of number of PhDs and SCA membership. The peer-review process can also act as a barrier to individuals from non-academic professions. While it is a challenge to track output affiliated with cultural resource management and agency roles (e.g., professional and industry literature, grey literature), a corpus of written output that is essential to our field, we employed a novel means of tracking un-refereed productivity (via the *Proceedings*) to give insight into the dynamics of how gender and occupation influence publication rates.

We suspect that the peer review gap will eventually be alleviated if certain structural aspects of the discipline change. For example, there may be greater gender equity if more women are hired in tenure-track positions, although given national trends indicating increasing numbers of non-tenure-track appointments nationwide, how this will ultimately play out remains to be seen. Similarly, if peer-reviewed publishing becomes a more supported and rewarded activity in compliance archaeology, more women, and a greater breadth of people from different occupations and backgrounds, will likely be represented in the pages of these journals.

The growing female contingent of SCA members (Fig 3) is an encouraging trend, for if enough women students maintain their membership and continue in the field, we may see the gender gap narrow in this new generation. Other researchers have noted increasingly even representation of women and men in younger age cohorts nationally as indicated by SAA membership (page 4 in [6]). Additionally, among social scientists in the Netherlands, younger women are publishing more and are cited more [3], indicating that the gender gap in publishing may be “aging out” in some settings. In any case, because *JCGBA* and *CA* each publish a relatively small number of articles and reports (about 10–15 per year), a handful of women authors could potentially make a very large impact on future trends and close the gender gap to more accurately reflect the overall participation of women in California and Great Basin archaeology.

In the future, we would like to track and recognize research output in more grey literature sources and track a broader range of participants. Our analysis of gender, unfortunately, cannot possibly account for non-gender-binary and non-gender conforming individuals. The lack of attention to queer/genderqueer/non-binary genders is a major component that is lacking in most equity studies, and is an issue that should be comprehensively addressed in future research on gender and publication rates in archaeology as well as in other disciplines. If journals began voluntary self-identification of gender upon paper submittal/ acceptance we would be able to better understand these patterns for people of all orientations and backgrounds. Similarly, it is also impossible to effectively study the publication and participation rates of underrepresented groups and self-identification of ethnicity may be a means of working this out in the future. There remains a need for archaeology as a discipline to be more intersectional and multi-vocal [60], and publication venues such as *Proceedings* have the potential to increase the representation of a variety of stakeholders and historically marginalized voices in addition to women and extra-academic professionals. Such considerations of identity and politics in archaeology are a necessary part of fulfilling our ethical responsibility as professionals [61]. While recognition and prestige influence an *individual*’s decision to publish (as well as the cost and benefit realities of an individual’s occupation), the larger message here is that the discipline can only benefit from the dissemination of different ideas and perspectives. Who ultimately controls the discourse of archaeology is precisely the point.

Archaeology has emerged as a field with equal or greater numbers of women participants compared to men. Like many other STEM disciplines there is a heavy contingent of extra-academic professionals; Today, nine out of ten archaeologists work in a growing industry that accounts for an estimated one billion dollars of annual spending (page 297 in [39]). While publication in peer-reviewed journals is a common benchmark for rating professional output, it is critical to recognize the realities of this professional landscape, and provide realistic means for disseminating the views and works of participants from a variety of backgrounds. Our case study provides a novel means of tracking the intricacies of participation and publication output over a long historic interval. Recognizing gaps in peer review publishing is an important step in alleviating these trends in the future. Additional studies will help us better understand whether these results are representative of other regions of North America, as well as on the national and international scales.

## Supporting information

S1 File **Text A.** Journal Summaries; **Table A.** National Archaeological Society Members, CRM firms, and PhD Programs from California and Great Basin States; **Table B.** Gender Composition of SCA Members (1967–2016); **Table C.** Gender representation of SCA membership categories, 2011 and 2016; **Table D.** Gender Composition of SCA Annual Meeting Contributors, 2011 and 2016; **Table E.** Paper and author counts of peer-reviewed and non-peer-reviewed journals; **Table F.** Gender of First and Total Authors, *JCA/ JCGBA* (1974–2016) (note: not published in 2003); **Table G.** Gender of First and Total Authors, *California Archaeology* (2009–2016); **Table H.** Gender of First and Total Authors, SCA *Proceedings* (1988–2016) (Note: *Proceedings* not published in 2002 or 2008); **Table I.** Gender of lead authors by occupational affiliation, *JCA/ JCGBA* (1974–2016); **Table J.** Gender of lead authors by occupational affiliation, *California Archaeology* (2009–2016); **Table K.** Gender of lead authors by occupational affiliation, SCA *Proceedings* (1988–2016).
(DOCX)
