# Supplementary material for: The peer review gap: A longitudinal case study of gendered publishing and occupational patterns in a female-rich discipline, Western North America (1974–2016)
Source: PLoS One. 2017 Nov 29;12(11):e0188403. doi: 10.1371/journal.pone.0188403 (PMC5706720; doi:10.1371/journal.pone.0188403)
Supplement: S1 File — Text A. Journal Summaries; Table A. National Archaeological Society Members, CRM firms, and PhD Programs from California and Great Basin States; Table B. Gender Composition of SCA Members (1967–2016); Table C. Gender representation of SCA membership categories, 2011 and 2016; Table D. Gender Composition of SCA Annual Meeting Contributors, 2011 and 2016; Table E. Paper and author counts of peer-reviewed and non-peer-reviewed journals; Table F. Gender of First and Total Authors, JCA/ JCGBA (1974–2016) (note: not published in 2003); Table G. Gender of First and Total Authors, California Archaeology (2009–2016); Table H. Gender of First and Total Authors, SCA Proceedings (1988–2016) (Note: Proceedings not published in 2002 or 2008); Table I. Gender of lead authors by occupational affiliation, JCA/ JCGBA (1974–2016); Table J. Gender of lead authors by occupational affiliation, California Archaeology (2009–2016); Table K. Gender of lead authors by occupational affiliation, SCA Proceedings (1988–2016). (DOCX) [file pone.0188403.s001.docx]

**Text A in S1 File. Journal Summaries**

***Journal of California Anthropology* (1974-1988)*/ Journal of California and Great Basin Anthropology* (1989-present)**

Over the past forty years, the Malki Museum, affiliated with the Morongo Indian Reservation in Banning, California, in cooperation with the University of California, Riverside, has published the *Journal of California Anthropology* (JCA) and *Journal of California and Great Basin Anthropology* (JCGBA). The *JCA* was introduced in 1974, spearheaded by Katherine Siva Saubel (President and Chairman of the Malki Museum Board of Directors) and an editorial staff including academics, Museum professionals, native Californians and others who sought to publish a “journal-format periodical devoted exclusively to native California anthropology in general, in which California anthropologists and Indians can exchange and discuss information of common concern” with the intent that the journal will serve the “thousands of living Indian people in California….as well as the professional needs of anthropologists working and studying with them” [1]:4. Thus, from its inception, the intent was to highlight the diversity of regional native peoples, and this would be facilitated by its diverse editorial staff and its solicitations of contributions from all anthropological subfields. The aspiration was that the journal would be of interest not only to local scholars, but also to world-wide anthropologists (especially hunter-gatherer specialists) and, with its general interest articles and essays, also attract lay audiences [1]:4-5.

In 1979, the *JCA* was renamed the *Journal of California and Great Basin Anthropology* (*JCGBA*), and though it expanded into publishing contributions on Great Basin anthropological research, the journal maintained editorial and publishing continuity. It is for this reason that we combined both titles in our analysis. The journal publishes articles on both Alta and Baja California, and the Great Basin, a culture area that spans eastern California, southeast Oregon, Nevada, Utah, western Colorado, northern Arizona, southern Idaho, western Wyoming.

Past Editors of the journal include 9 men and two women from academic and compliance archaeology backgrounds who are assisted by an editorial board. The Journal has followed a single blind review process, (referees remain anonymous, but the name and affiliation of authors are revealed to reviewers), although the journal is shifting to a double-blind model (Adrian Whitaker, Personal Communication 2016). *JCA/ JCGBA* is published biannually (except in 1983 when both issues were published in a single volume), and has been published every year since its inception except 2003. The Malki Museum’s website provides information about the journal at: <http://www.malkimuseum.org/journals.htm>. Articles published before 2009 are available online at: <http://www.escholarship.org/uc/ucmercedlibrary_jcgba>.

**Society for California Archaeology *Proceedings* (1988-present)**

First published in 1988, the Society for California (SCA) Archaeology *Proceedings* provides a publication forum for papers presented at the SCA annual meeting. As stated on the SCA website, the intent of the *Proceedings* is to disseminate research promptly, and to “serve as a forum for all who participate in this annual celebration of California’s rich and diverse past: archaeologists and Native Americans, avocationalists and professionals, professors and students, historians and prehistorians, and government staff and the public they serve.” The journal has been published annually except in 2001 and 2008. Most articles highlight the archaeology of California, but a substantial number come from Baja California, Mexico, and from neighboring states and even international venues: <https://scahome.org/sca-publications/articles-of-the-sca-proceedings/?sort=2>

Publication in *Proceedings* is open to anyone who presented a paper at the previous year’s SCA annual meeting unless the paper has appeared in other publications (see <https://scahome.org/sca-publications/proceeding-submissions/>). It is a non-peer-reviewed publication, meaning that papers are not sent to referees for review, although the editors correct manuscripts for style and grammar, standardize formatting and graphics, and check to ensure that contributions conform to the SCA’s Bylaws and Ethical Guidelines. However, it is the authors’ responsibility to ensure the content of articles is accurate, and that they have the legal right to publish figures. Since its inception the SCA Proceedings has been edited by a committee of between two and five. Editorial committees have historically included roughly equal numbers of men and women, and editors have varied occupational affiliations (academia, CRM, and agency archaeology).

Prior to 2009, the *Proceedings* was published in print and was sent to all members of the SCA. As such, its circulation was limited to the SCA membership and subscribing libraries. In 2009, the *Proceedings* began an online-only format, a shift that has dramatically increased the visibility of the papers it publishes as well as the speed of publication. The *Proceedings* is an “open access” publication available at <https://scahome.org/sca-publications/articles-of-the-sca-proceedings/>, meaning that it does not require a paid subscription so that anyone with Internet access can download its content.

***California Archaeology* (*CA*) (2009-present)**

In 2009, several important and related events occur in SCA publishing: the Society published the first issue of its flagship journal, *California Archaeology,* and it moved the SCA *Proceedings* and quarterly *Newsletter* to online-only formats. Thus, for the first time, the Society offered a peer-reviewed venue (*California Archaeology*) to compliment the much older *JCGBA*. Concurrent with these changes was a major reshuffling of content. For example, many items formerly contained in the *Newsletter* were redirected to *California Archaeology*, including longer articles and reports (for the peer-reviewed section of *CA*), short contributions and news items (for the non-peer-reviewed section, News and Notes), and obituaries.

*California Archaeology* includes articles and reports on the prehistory and history of the California culture area, which includes the state of California and southwest Oregon (often referred to as “Alta California by archaeologists), as well as Baja, Mexico (“Baja California”). Unlike the four field approach of *JCGBA*, *CA* is explicitly dedicated to archaeology; According to the SCA website, ethnohistoric, ethnographic and paleoenvironmental studies “will be considered only if they make explicit connections to or have clear implications for the material record” and “Papers dealing with the recent past (<50 years) will be given lower priority for publication.” Papers are submitted to a single-blind review process (referees remain anonymous, but the name and affiliation of authors are revealed to reviewers). Since its inception the journal has been edited by Terry Jones. He is a Professor of Anthropology at Cal Poly, San Luis Obispo.

In contrast with *JCGBA*, all of *CA*’s contents are available electronically; however, the content is behind a paywall and only open to individual or institutional subscribers. *CA* was initially published by Left Coast Press, and is currently published by Taylor and Francis Online: <http://www.tandfonline.com/loi/ycal20>. Information about the journal is also available on the SCA’s website: <https://scahome.org/sca-publications/california-archaeology-the-sca-journal/>.

**Table A in S1 File. National Archaeological Society Members, CRM firms, and PhD Programs from California and Great Basin States**

|  | ***Register of Professional Archaeologists Members 2017**** | | ***Society for American Archaeology Members 2015***** | | ***CRM Firms/Organizations 2017****** | | ***PhD Programs (Archaeology Focus)******* | |
| --- | --- | --- | --- | --- | --- | --- | --- | --- |
|  | ***Total in USA = 2556*** | | ***Total in USA = 2120*** | | ***Total in USA = 2210*** | | ***Total in USA = 75*** | |
| **State** | **Count** | **% of Total** | **Count** | **% of total** | **Count** | **% of total** | **Count** | **% of total** |
| **CA** | 451 | 17.6% | 311 | 14.7% | 57 | 2.6% | 6 | 8.0% |
| **AZ** | 120 | 4.7% | 145 | 6.8% | 47 | 2.1% | 2 | 2.7% |
| **CO** | 96 | 3.8% | 97 | 4.6% | 52 | 2.4% | 2 | 2.7% |
| **ID** | 33 | 1.3% | 27 | 1.3% | 48 | 2.2% | 0 | 0.0% |
| **NV** | 67 | 2.6% | 46 | 2.2% | 49 | 2.2% | 2 | 2.7% |
| **OR** | 102 | 4.0% | 60 | 2.8% | 45 | 2.0% | 2 | 2.7% |
| **UT** | 46 | 1.8% | 36 | 1.7% | 47 | 2.1% | 0 | 0.0% |
| **WY** | 29 | 1.1% | 29 | 1.4% | 44 | 2.0% | 1 | 1.3% |
| **Notes:** Data above reflect state of residence. We list sites that are entirely or partially part of the California and Great Basin culture areas (the subject of the journal articles covered in this article).  *Register of Professional Archaeologists (RPA) Membership by State Directory available at: http://rpanet.org/page/20172018Directory; Accessed 11.4.2017. | | | | | | | | |
| **Society for American Archaeology (SAA) 2015 Membership Needs Assessment Survey. Members only survey available at: http://ecommerce.saa.org/saa/Member/For_Members/2015_SAA_Needs_Assessment_Survey.  ***American Cultural Resources Association (ACRA) Membership by State Directory available at: http://www.acra-crm.org/member-directory; Accessed 11.5.2017.  ****American Anthropological Association AnthroGuide, Limited to Current Anthroguide listers; AnthroGuide available at: https://secure.americananthro.org/eweb/DynamicPage.aspx?Site=AAAWeb&WebKey=cc464c00-c91e-497c-b51a-7e0d27b96daa; Accessed 11.5.2017. | | | | | | | | |

**Table B in S1 File. Gender Composition of SCA Members (1967-2016)**

|  | **Male** | **Female** | **Unknown** |
| --- | --- | --- | --- |
| **1967** | 21 | 5 | 0 |
| **1971** | 238 | 126 | 16 |
| **1981** | 257 | 202 | 37 |
| **1991** | 281 | 247 | 26 |
| **2000** | 181 | 197 | 16 |
| **2011** | 533 | 623 | 20 |
| **2016** | 569 | 606 | 18 |

**Table C in S1 File. Gender representation of SCA membership categories, 2011 and 2016**

|  | ***2011*** | | | ***2016*** | | |
| --- | --- | --- | --- | --- | --- | --- |
| **Membership Category** | **Male** | **Female** | **Unknown** | **Male** | **Female** | **Unknown** |
| Student | 102 | 184 | 6 | 78 | 147 | 7 |
| Regular | 278 | 294 | 12 | 278 | 313 | 8 |
| Contributing | 3 | 2 | 1 | 11 | 3 | - |
| Lifetime | 43 | 37 | - | 34 | 32 | - |
| Spouse | 5 | 18 | - | 6 | 12 | - |
| Admin | 3 | 2 | - | - | - | - |
| Senior | 92 | 83 | 1 | 139 | 90 | 3 |
| Senior Lifetime | 7 | 3 | - | 16 | 8 | - |
| Contributing Senior | - | - | - | 7 | 1 | - |
| **Grand Total** | **533** | **623** | **20** | **569** | **606** | **18** |

**Table D in S1 File. Gender Composition of SCA Annual Meeting Contributors, 2011 and 2016**

|  | ***2011*** | | | ***2016*** | | |
| --- | --- | --- | --- | --- | --- | --- |
| **Presentation type** | **Male** | **Female** | **Unknown** | **Male** | **Female** | **Unknown** |
| **Paper** | 132 | 89 | - | 202 | 188 | - |
| **Poster** | 12 | 23 | - | 49 | 60 | 4 |
| **Forum** | 7 | 14 | - | 1 | 8 | - |

**Table E in S1 File. Paper and author counts of peer-reviewed and non-peer reviewed journals**

|  | ***PEER-REVIEWED*** | | ***NOT PEER-REVIEWED*** |
| --- | --- | --- | --- |
|  | ***JCA/JCGBA*** | ***CA Archaeology*** | **SCA *Proceedings*** |
|  | (1974-2016) | (2009-2016) | (1988-2016) |
| TOTAL # of papers | 710 | 85 | 804 |
| TOTAL # of authors | 1179 | 204 | 1234 |
| Range (# of papers per year) | 8 to 28 | 9 to 14 | 11 to 95 |
| Mean # of papers (per year) | 16.9 | 10.6 | 31.9 |
| Median # of papers (per year) | 17.0 | 10.0 | 27.0 |
| Standard deviation (per year) | 5.1 | 1.9 | 17.3 |
|  |  |  |  |

**Notes:** *JCA/JCBA* was not published in 2003; *Proceedings* was not published in 2001, 2008.

**Table F in S1 File. Gender of First and Total Authors, *JCA/ JCGBA* (1974-1976) (note: not published in 2003)**

| **Publication year** | **Female 1st/ Sole** | **Male 1st/Sole** | **Unknown 1st/ Sole** | **Total Papers** | **%Female 1st/ Sole** | **Total Female Authors** | **Total Male Authors** | **Total Unknown Authors** | **Total Authors** | **%Female Total Authors** |
| --- | --- | --- | --- | --- | --- | --- | --- | --- | --- | --- |
| 1974 | 5 | 14 | 0 | 19 | 26.3% | 5 | 15 | 0 | 20 | 25.0% |
| 1975 | 5 | 18 | 0 | 23 | 21.7% | 5 | 18 | 0 | 23 | 21.7% |
| 1976 | 3 | 16 | 0 | 19 | 15.8% | 5 | 21 | 0 | 26 | 19.2% |
| 1977 | 5 | 19 | 0 | 24 | 20.8% | 7 | 25 | 0 | 32 | 21.9% |
| 1978 | 6 | 18 | 0 | 24 | 25.0% | 6 | 25 | 0 | 31 | 19.4% |
| 1979 | 2 | 20 | 0 | 22 | 9.1% | 7 | 27 | 1 | 35 | 20.0% |
| 1980 | 7 | 21 | 0 | 28 | 25.0% | 8 | 25 | 0 | 33 | 24.2% |
| 1981 | 4 | 20 | 0 | 24 | 16.7% | 9 | 27 | 0 | 36 | 25.0% |
| 1982 | 4 | 18 | 1 | 23 | 17.4% | 6 | 27 | 1 | 34 | 17.6% |
| 1983 | 3 | 18 | 1 | 22 | 13.6% | 3 | 21 | 1 | 25 | 12.0% |
| 1984 | 3 | 15 | 0 | 18 | 16.7% | 3 | 19 | 0 | 22 | 13.6% |
| 1985 | 3 | 19 | 0 | 22 | 13.6% | 7 | 24 | 1 | 32 | 21.9% |
| 1986 | 6 | 15 | 1 | 22 | 27.3% | 9 | 27 | 1 | 37 | 24.3% |
| 1987 | 6 | 12 | 0 | 18 | 33.3% | 7 | 22 | 0 | 29 | 24.1% |
| 1988 | 3 | 18 | 0 | 21 | 14.3% | 6 | 25 | 0 | 31 | 19.4% |
| 1989 | 2 | 11 | 0 | 13 | 15.4% | 5 | 13 | 1 | 19 | 26.3% |
| 1990 | 9 | 8 | 0 | 17 | 52.9% | 14 | 13 | 0 | 27 | 51.9% |
| 1991 | 5 | 14 | 0 | 19 | 26.3% | 6 | 22 | 0 | 28 | 21.4% |
| 1992 | 2 | 15 | 0 | 17 | 11.8% | 6 | 25 | 0 | 31 | 19.4% |
| 1993 | 3 | 10 | 0 | 13 | 23.1% | 4 | 16 | 0 | 20 | 20.0% |
| 1994 | 1 | 10 | 1 | 12 | 8.3% | 5 | 15 | 1 | 21 | 23.8% |
| 1995 | 5 | 10 | 0 | 15 | 33.3% | 9 | 18 | 0 | 27 | 33.3% |
| 1996 | 3 | 12 | 0 | 15 | 20.0% | 9 | 26 | 1 | 36 | 25.0% |
| 1997 | 2 | 11 | 0 | 13 | 15.4% | 5 | 22 | 0 | 27 | 18.5% |
| 1998 | 4 | 10 | 0 | 14 | 28.6% | 8 | 17 | 0 | 25 | 32.0% |
| 1999 | 3 | 12 | 0 | 15 | 20.0% | 3 | 20 | 0 | 23 | 13.0% |
| 2000 | 2 | 23 | 0 | 25 | 8.0% | 7 | 38 | 0 | 45 | 15.6% |
| 2001 | 1 | 7 | 0 | 8 | 12.5% | 2 | 11 | 0 | 13 | 20.0% |
| 2002 | 3 | 14 | 0 | 17 | 17.6% | 6 | 24 | 0 | 30 | 15.4% |
| 2003 | - | - | - | - | - | - | - | - | - | - |
| 2004 | 3 | 5 | 0 | 8 | 37.5% | 5 | 8 | 0 | 13 | 38.5% |
| 2005 | 4 | 11 | 0 | 15 | 26.7% | 7 | 28 | 0 | 35 | 20.0% |
| 2006 | 1 | 9 | 0 | 10 | 10.0% | 3 | 10 | 0 | 13 | 23.1% |
| 2007 | 4 | 7 | 0 | 11 | 36.4% | 6 | 12 | 0 | 18 | 33.3% |
| 2008 | 3 | 7 | 0 | 10 | 30.0% | 4 | 15 | 0 | 19 | 21.1% |
| 2009 | 3 | 6 | 0 | 9 | 33.3% | 5 | 16 | 0 | 22 | 22.7% |
| 2010 | 5 | 7 | 0 | 12 | 41.7% | 9 | 10 | 0 | 19 | 47.4% |
| 2011 | 5 | 8 | 0 | 13 | 38.5% | 6 | 21 | 0 | 27 | 22.2% |
| 2012 | 3 | 9 | 0 | 12 | 25.0% | 11 | 19 | 0 | 30 | 36.7% |
| 2013 | 5 | 9 | 0 | 14 | 35.7% | 11 | 20 | 0 | 31 | 35.5% |
| 2014 | 6 | 10 | 0 | 16 | 37.5% | 16 | 24 | 0 | 40 | 40.0% |
| 2015 | 5 | 13 | 0 | 18 | 27.8% | 11 | 34 | 0 | 45 | 24.4% |
| 2016 | 9 | 11 | 0 | 20 | 45.0% | 18 | 32 | 0 | 50 | 36.0% |
| **Grand Total** | **166** | **540** | **4** | **710** | **23.4%** | **294** | **877** | **8** | **1179** | **24.9%** |
|  | 23.4% | 76.1% | 0.6% | 100.0% |  | 24.9% | 74.4% | 0.7% | 100.0% |  |

**Table G in S1 File. Gender of First and Total Authors, *California Archaeology* (2009-2016)**

| **Publication year** | **Female 1st/ Sole** | **Male 1st/Sole** | **Total Papers** | **%Female 1st/ Sole** | **Total Female authors** | **Total Male Authors** | **Total Authors** | **%Female Total Authors** |
| --- | --- | --- | --- | --- | --- | --- | --- | --- |
| 2009 | 0 | 13 | 13 | 0.0% | 2 | 18 | 20 | 10.0% |
| 2010 | 2 | 7 | 9 | 22.2% | 2 | 13 | 15 | 13.3% |
| 2011 | 2 | 9 | 11 | 18.2% | 5 | 11 | 16 | 31.3% |
| 2012 | 2 | 8 | 10 | 20.0% | 5 | 15 | 20 | 25.0% |
| 2013 | 3 | 11 | 14 | 21.4% | 15 | 28 | 43 | 34.9% |
| 2014 | 3 | 6 | 9 | 33.3% | 11 | 15 | 26 | 42.3% |
| 2015 | 4 | 5 | 9 | 44.4% | 18 | 18 | 36 | 50.0% |
| 2016 | 2 | 8 | 10 | 20.0% | 11 | 17 | 28 | 39.3% |
| **Grand Total** | **18** | **67** | **85** | **21.2%** | **69** | **135** | **204** | **33.8%** |
|  | 21.2% | 78.8% |  |  | 33.8% | 66.2% |  |  |

**Table H in S1 File. Gender of First and Total Authors, SCA *Proceedings* (1988-2016)** (Note*: Proceedings* not published in 2002 or 2008)

|  | **Female 1st/Sole** | **Male 1st/Sole** | **Unknown 1st/Sole** | **Total Papers** | **%Female 1st/Sole** | **Total Female Authors** | **Total Male Authors** | **Total Unknown Authors** | **Total Authors** | **%Female Total Authors** |
| --- | --- | --- | --- | --- | --- | --- | --- | --- | --- | --- |
| 1988 | 12 | 14 | 0 | 26 | 46.2% | 16 | 18 | 0 | 34 | 47.1% |
| 1989 | 6 | 8 | 0 | 14 | 42.9% | 8 | 9 | 0 | 17 | 47.1% |
| 1990 | 5 | 18 | 0 | 23 | 21.7% | 8 | 20 | 0 | 28 | 28.6% |
| 1991 | 5 | 12 | 0 | 17 | 29.4% | 7 | 17 | 0 | 24 | 29.2% |
| 1992 | 8 | 11 | 0 | 19 | 42.1% | 11 | 14 | 0 | 25 | 44.0% |
| 1993 | 15 | 21 | 0 | 36 | 41.7% | 17 | 32 | 1 | 50 | 34.0% |
| 1994 | 7 | 16 | 0 | 23 | 30.4% | 8 | 21 | 0 | 29 | 27.6% |
| 1995 | 4 | 17 | 0 | 21 | 19.0% | 4 | 21 | 0 | 25 | 16.0% |
| 1996 | 16 | 29 | 0 | 45 | 35.6% | 21 | 36 | 0 | 57 | 36.8% |
| 1997 | 11 | 16 | 0 | 27 | 40.7% | 16 | 19 | 0 | 35 | 45.7% |
| 1998 | 8 | 14 | 0 | 22 | 36.4% | 13 | 16 | 0 | 29 | 44.8% |
| 1999 | 11 | 20 | 0 | 31 | 35.5% | 12 | 26 | 0 | 38 | 31.6% |
| 2000 | 11 | 22 | 0 | 33 | 33.3% | 14 | 29 | 0 | 43 | 32.6% |
| 2001 | - | - | - | - | - | - | - | - | - | - |
| 2002 | 1 | 10 | 0 | 11 | 9.1% | 6 | 15 | 0 | 21 | 28.6% |
| 2003 | 5 | 25 | 0 | 30 | 16.7% | 9 | 31 | 0 | 40 | 22.5% |
| 2004 | 18 | 31 | 0 | 49 | 36.7% | 26 | 52 | 0 | 78 | 33.3% |
| 2005 | 16 | 26 | 0 | 42 | 38.1% | 23 | 35 | 0 | 58 | 39.7% |
| 2006 | 12 | 27 | 0 | 39 | 30.8% | 20 | 48 | 0 | 68 | 29.4% |
| 2007 | 4 | 13 | 0 | 17 | 23.5% | 13 | 28 | 0 | 41 | 31.7% |
| 2008 | - | - | - | - | - | - | - | - | - | - |
| 2009 | 48 | 47 | 0 | 95 | 50.5% | 76 | 84 | 0 | 160 | 47.5% |
| 2010 | 16 | 9 | 0 | 25 | 64.0% | 24 | 22 | 0 | 46 | 52.2% |
| 2011 | 8 | 12 | 1 | 21 | 38.1% | 18 | 24 | 1 | 43 | 41.9% |
| 2012 | 15 | 13 | 0 | 28 | 53.6% | 24 | 24 | 0 | 48 | 50.0% |
| 2013 | 14 | 13 | 0 | 27 | 51.9% | 21 | 19 | 0 | 40 | 52.5% |
| 2014 | 16 | 21 | 0 | 37 | 43.2% | 25 | 38 | 0 | 63 | 39.7% |
| 2015 | 12 | 7 | 0 | 19 | 63.2% | 18 | 15 | 0 | 33 | 54.5% |
| 2016 | 9 | 18 | 0 | 27 | 33.3% | 23 | 37 | 1 | 61 | 37.7% |
| **Grand Total** | **313** | **490** | **1** | **804** | **38.9%** | **481** | **750** | **3** | **1234** | **39.0%** |
|  | 38.9% | 60.9% | 0.1% | 100.0% |  | 39.0% | 60.8% | 0.2% | 100.0% |  |

**Table I in S1 File. Gender of lead authors by occupational affiliation, *JCA/ JCGBA* (1974-2016)**

|  | ***Lead authors*** | | |  |
| --- | --- | --- | --- | --- |
| **Affiliation** | **Female** | **Male** | **Unknown** | **Total** |
| **Academic** | 103 | 340 | 1 | 444 |
| **Agency** | 16 | 44 | 1 | 61 |
| **Independent** | 24 | 47 | 1 | 72 |
| **Museum** | 3 | 34 | 0 | 37 |
| **Other** | 3 | 13 | 0 | 16 |
| **Private sector (CRM)** | 15 | 61 | 1 | 77 |
| **Tribal organization** | 2 | 1 | 0 | 3 |
| **Grand Total** | 166 | 540 | 4 | 710 |

**Table J in S1 File. Gender of lead authors by occupational affiliation, *California Archaeology* (2009-2016)**

|  | ***Lead authors*** | |  |
| --- | --- | --- | --- |
| **Affiliation** | **Female** | **Male** | **Total** |
| **Academic** | 11 | 32 | 43 |
| **Agency** | 2 | 8 | 10 |
| **Independent** | 0 | 2 | 2 |
| **Museum** | 2 | 2 | 4 |
| **Private sector (CRM)** | 2 | 22 | 24 |
| **Tribal organization** | 0 | 1 | 1 |
| **Grand Total** | **17** | **67** | **84** |

**Table K in S1 File. Gender of lead authors by occupational affiliation, SCA *Proceedings* (1988-2016)**

|  | ***Lead authors*** | |  |
| --- | --- | --- | --- |
| **Affiliation** | **Female** | **Male** | **Total** |
| **Academic** | 109 | 193 | 302 |
| **Agency** | 58 | 107 | 165 |
| **Independent** | 25 | 28 | 53 |
| **Museum** | 6 | 11 | 17 |
| **Other** | 25 | 30 | 55 |
| **Private sector (CRM)** | 89 | 120 | 209 |
| **Tribal organization** | 1 | 1 | 2 |
| **Grand Total** | **313** | **490** | **803** |

**References**

1.	Kearney M. Introductory. Journal of California Anthropology. 1974;1(1):4-5.
